# Efficiency of Rapid Whole-Genome Sequencing in Pediatric Intensive Care Units: Experience in a Diverse Population

**DOI:** 10.3390/ijms27209262

**Published:** 2026-10-19

**Authors:** Christopher Simotas, Kaitlyn Kurtz, Aline Sandouk, Christina Botti, Aadya Chawla, Christina Kresge, Katarzyna Ellsworth, Deepti Purohit, Meera Kale, Milen Velinov

**Affiliations:** 1 Division of Genetics, Department of Pediatrics, Rutgers Robert Wood Johnson Medical School, New Brunswick, NJ 08901, USA; simotach@rwjms.rutgers.edu (C.S.);; 2 Division of General Pediatrics and Community Health, Children’s National Hospital, Washington, DC 20010, USA; 3 Department of Pediatrics, The George Washington University School of Medicine and Health Sciences, Washington, DC 20052, USA; 4 The Unterberg Children’s Hospital, Monmouth Medical Center, Long Branch, NJ 07740, USA; 5 Rutgers New Jersey Medical School, Newark, NJ 07103, USA; 6 Rady Children’s Institute for Genomic Medicine, San Diego, CA 92123, USA; kellsworth@rchsd.org; 7 Department of Neonatology, Department of Pediatrics, Childrens’ Hospital of New Jersey, Beth Israel Medical Center, Newark, NJ 07112, USA; deepti.purohit@rwjbh.org; 8 General Pediatrics, Department of Pediatrics, Rutgers Robert Wood Johnson Medical School, New Brunswick, NJ 08901, USA

**Keywords:** rapid whole genome sequencing, NICU, PICU, next generation sequencing, clinical genetics

## Abstract

Single-gene disorders are a common cause of infant and young children’s mortality in intensive care units. Many of these genetic disorders have available treatments that may slow disease progression and improve patients’ quality of life. However, clinicians often identify the underlying genetic etiology late in the disease course, when available treatments cannot reverse disease progression. Rapid whole-genome sequencing (RWGS) is increasingly used for early diagnosis of patients with rare disorders. It remains unclear which clinical indications are most efficient for providing actionable genetic diagnoses. It is also not well studied how efficient RWGS is in diverse populations where population genomic data is less abundant. We present RWGS results over a three-year period from four medical centers within a large hospital system in the State of New Jersey, which has one of the most diverse populations in the United States. The patients were referred to RWGS testing from the NICU and PICU units for various severe/life-threatening manifestations. All patients were tested at one laboratory center using the same technology for genome sequencing and analysis, as described, to eliminate possible interlaboratory variation in test efficiency. We identified a causative genetic etiology in 19 (36.5%) of the 52 tested individuals. We observed the highest yield of positive diagnoses in patients with unexplained failure to thrive (FTT). The genetic diagnoses were actionable for disease-specific, supportive, or progression-slowing targeted treatments, better-informed genetic counseling, or modifying the level of care for most positive individuals. Our study provides information on the test efficiency of RWGS in diverse populations and compares testing efficiency across leading referral indications. The study may help guide referral decisions for such testing.

## 1. Introduction

The Online Mendelian Inheritance in Man (OMIM) database lists over 7600 single-gene disease phenotypes associated with over 5000 single genes. Since 2014, the OMIM database has added approximately 300 newly discovered, primarily single-gene disorders annually [1]. Approximately 70% of all single-gene disorders have childhood onset, and timely diagnosis and management may improve long-term clinical outcomes if started early. Early diagnosis may also help determine the most appropriate management approach and support family planning. Rapid whole-genome sequencing (RWGS) is becoming a standard of care for sick newborns and young children in intensive care units because of the high prevalence of genetic disorders in this patient group [2,3]. However, the indications for such testing and its efficiency across different population groups remain poorly defined. Previous studies did not analyze the efficiency of RWGS for a specific indication [2,3,4]. We report RWGS evaluation results in 52 individuals across four hospital centers in New Jersey. Although our study used similar indications for testing, it included a highly diverse patient population. In addition, this study provides limited pilot data on the efficiency of achieving genetic diagnoses across different referral indications and details patient outcomes among individuals with positive testing. This study may help prioritize clinical indications for RWGS.

## 2. Results

### Proportion of Diagnosed Individuals

**Figure 1:**
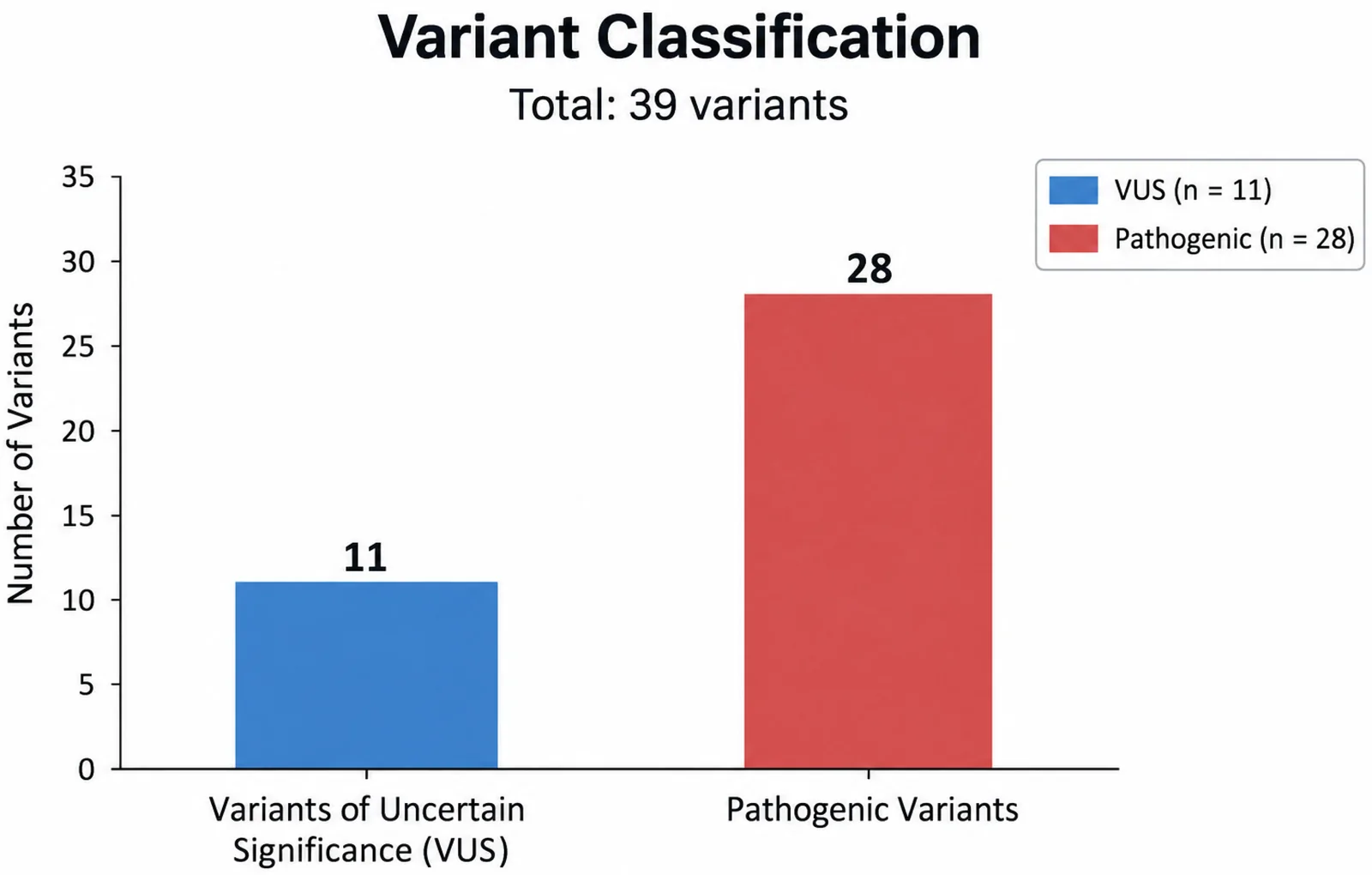
Overall proportion of pathogenic (causative) variants and variants of unknown significance.

The rapid WGS identified the causative chromosomal or single-gene variants in 19 out of the 52 referred-for-testing patients (36.5%). Figure 1 shows the proportion of pathogenic variants and variants of uncertain significance. 

Table 1 shows the proportion of positive results for each demographic group.

Among Black and Hispanic/Latino participants, the observed diagnostic success was lower than average. However, because of the small sample size, these differences were not statistically significant using a chi-square test. This suggests that differences may reflect the less abundant genomic data available for the populations studied.

**Figure 2:**
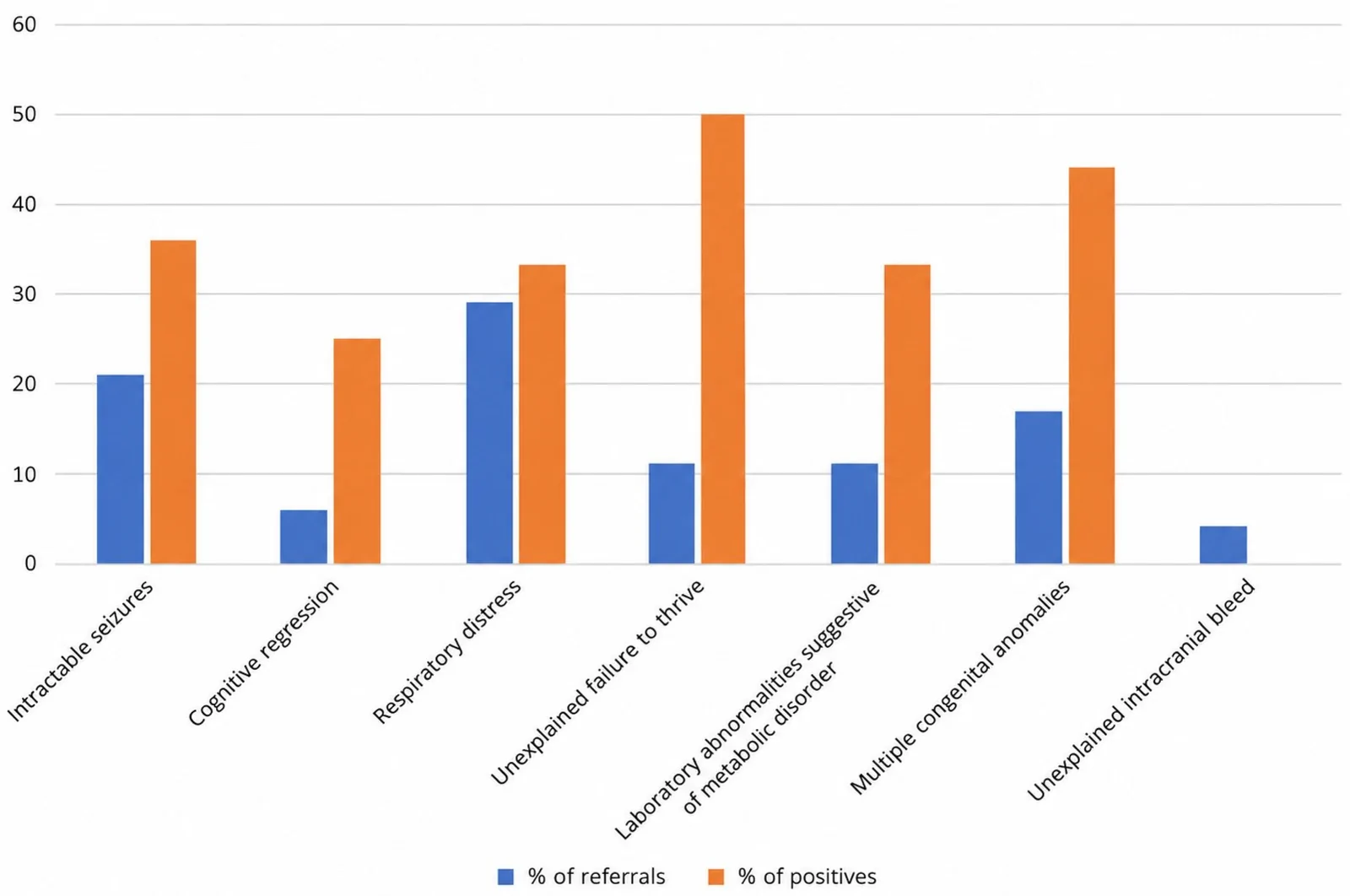
Indications for referrals and proportions of positives.

Figure 2 lists the leading manifestations triggering RWGS referrals and the corresponding proportion of positive findings for every referral group.

Table 2 lists the positive individuals, the corresponding leading reason for testing referral, the test finding, and the resulting change in management.

**Table 2:** Positive patients and diagnosis-related changes in management.

Patient ID	Age at the Time of Diagnosis	Gender	Gene	Inheritance	Clinical Diagnosis	Change in Management
1-7	5.5 years	female	KIAA0586	biparental	Joubert syndrome 23	1,2
1-10	7 months	male	ATP1A2	heterozygous, de novo	Developmental and epileptic encephalopathy 98	1,2
1-13	4 weeks	female	MMUT	biparental	Methylmalonic acidemia	1,2,3
1-16	17.5 years	female	POLG	biparental	POLG-associated mitochondrial disorder	1,2
1-21	1 week	male	HIBCH	biparental	3-hydroxyisobutyryl-CoA hydrolase deficiency	1
1-22	4.5 years	female	NF1and deletion 22q11.2 (maternal)	NF1-paternal, del 22a11 maternal	Neurofibromatosis, Velo-cardio-facial syndrome	1,2
1-24	3 months	male	ATP7A	heterozygous, de novo	Menkes disease	1,2,3
1-25	5 days	female	16q24.1 deletion including FOXF1	heterozygous, de novo	Alveolar capillary dysplasia	1,4
1-27	2.5 months	female	UBE3B	biparental	Kaufman oculocerebral syndrome	1,2
1-28	2 weeks	female	4.4 kb mitochondrial deletion	de novo	Mitochondrial deletion syndrome	1,2
1-30	6 weeks	male	POLE	biparental	IMAGe-I syndrome	1
1-31	6 months	female	ANTXR2	biparental	Hyaline fibromatosis syndrome	1,2,4
2-1	6 days	male	MITF	heterozygous, de novo	MITF-associated disorder	1,2
2-2	3 weeks	female	PORCN	heterozygous, de novo	PORCN-associated developmental disorder	1,2
2-7	3 weeks	female	RYR1	heterozygous, not maternal	RYR1-related disorder	1,2
3-9	2 months	male	COL9A1	biparental	Stickler syndrome, type V	1,2
3-2	2 weeks	male	3.6 Mb deletion 9p24.3p24.2	de novo	9p deletion syndrome	1,2
4-2	3 weeks	male	GNAS	de novo	Albright osteodystrophy syndrome	1,2
4-5	2 weeks	male	chr 15 q11.2q13.1	de novo	Prader–Willi syndrome	1,2,3

Legend: 1. helped inform family planning; 2. helped initiate disease-specific supportive management; 3. helped initiate targeted treatment; 4. led to change in level of care.

Cases 1–24 and 1–31 from Table 3 were previously reported [5,6].

Table 3 shows the HGVS classification of the variants.

**Table 3:** HGVS classification of variants.

Participant ID	HGVS Variant
1-7	NM_001244189.2(KIAA0586):c.428del (p.Arg143fs), NM_001244189.2(KIAA0586):c.1214C>G (p.Ser405Ter)
1-10	NM_000702.4(ATP1A2):c.2936C>T (p.Pro979Leu), NM_004572.4(PKP2):c.1613G>A (p.Trp538Ter)
1-13	NM_000255.4(MMUT): c.785G>A (p.Ser262Asn), NM_000255.4(MMUT):c.1415C>T (p.Ala472Val)
1-16	NM_002693.3(POLG):c.1399G>A(p.Ala467Thr), NM_002693.3(POLG):c.2243G>C(p.Trp748Ser)
1-29	NM_014362.4(HIBCH):c.129dup (p.Gly44fs), NM_014362.4(HIBCH):c.830T>A (p.Val277Glu)
1-22	NM_001042492.3(NF1):c.1641+1G>T, NC_000022.11:g.20732224_21466459del
1-24	NM_000052.7(ATP7a):c.870_871del(p.Ser293fs)
1-25	NC_000016.10:g.86400152_86647774del
1-27	NM_130466.4(UBE3B):c.1972C>T (p.Arg658Ter)
1-28	NC_012920.1:m.10952_15361del
1-30	NM_006231.4(POLE):c.3275G>C (p.Arg1092Thr), NM_006231.4(POLE):c.286-12G>A
1-31	NM_058172.6(ANTXR2):c.652T>C (p.Cys218Arg)
2-1	NM_000287.4(PEX6):c.232C>T (p.Arg78Cys), NM_000248.4:(MITF):c.731G>A (p.GLy244Glu)
2-2	NM_203475.3(PORCN):c.845+2T>G
2-7	NM_000540.3(RYR1):c.10348-6C>G, NM_000540.3(RYR1):c.14524G>A (p.Val4842Met)
3-2	NC_000009.12:g.204085_3810555del
3-9	NM_001852.4(COL9A2):c.1235del (p.Gly412fs)
4-2	NM_000516.7(GNAS):c.139G>A (p.Gly47Ser)
4-5	NC_000015.10:g.23615821_28563349del

## 3. Discussion

The diagnostic yield in our cohort was comparable to that reported in previous studies [2,7,8,9,10].

Minority groups were well represented in our study, but the observed diagnostic yield was lower in these demographic groups, possibly because genomic data for these populations is more limited. These differences were not statistically significant, likely because of the small sample size. This study further emphasizes the need to enroll non-European population groups in genomic analyses [11].

Interestingly, the referrals for unexplained failure to thrive and multiple congenital anomalies had the highest proportion of genomic diagnoses. Again, we could not achieve statistical significance for these differences because of the limited sample size. Seizures and respiratory distress, the most common reasons for referral, were also associated with high diagnostic yield.

For all individuals with genomic diagnoses, the test supported better family planning (recognizing recurrence risk for future pregnancies and the ability to conduct prenatal or pre-implantation diagnosis for the identified genetic condition) and disease-specific supportive care, including surveillance for potential future disease manifestations. For three of these patients, the test helped provide disease-specific targeted treatment to slow disease progression. For the patient with ID 1-13, the test helped implement specific metabolic management for methylmalonic acidemia; for the patient with ID 1-24, the test helped implement specific copper histidine therapy for Menkes disease; for the patient with ID 4-5, the test helped initiate growth hormone therapy. For two positive patients (IDs 1-25 and 1-31), the test helped transition to palliative care because of the unfavorable disease prognosis. Patients with VUS or a negative test were offered reanalysis every two years after the initial testing.

A major limitation of the study is the small number of patients studied. Nevertheless, this study demonstrates the value of RWGS used early in the diagnostic process. Early diagnosis of rare disorders may help provide targeted treatment in a significant proportion of cases. In addition, our study suggests that larger-cohort studies are needed to better address testing efficiency across different population groups and specific referral reasons.

This study was approved by the Rutgers University IRB.

## 4. Materials and Methods

### 4.1. Patients

**Figure 3:**
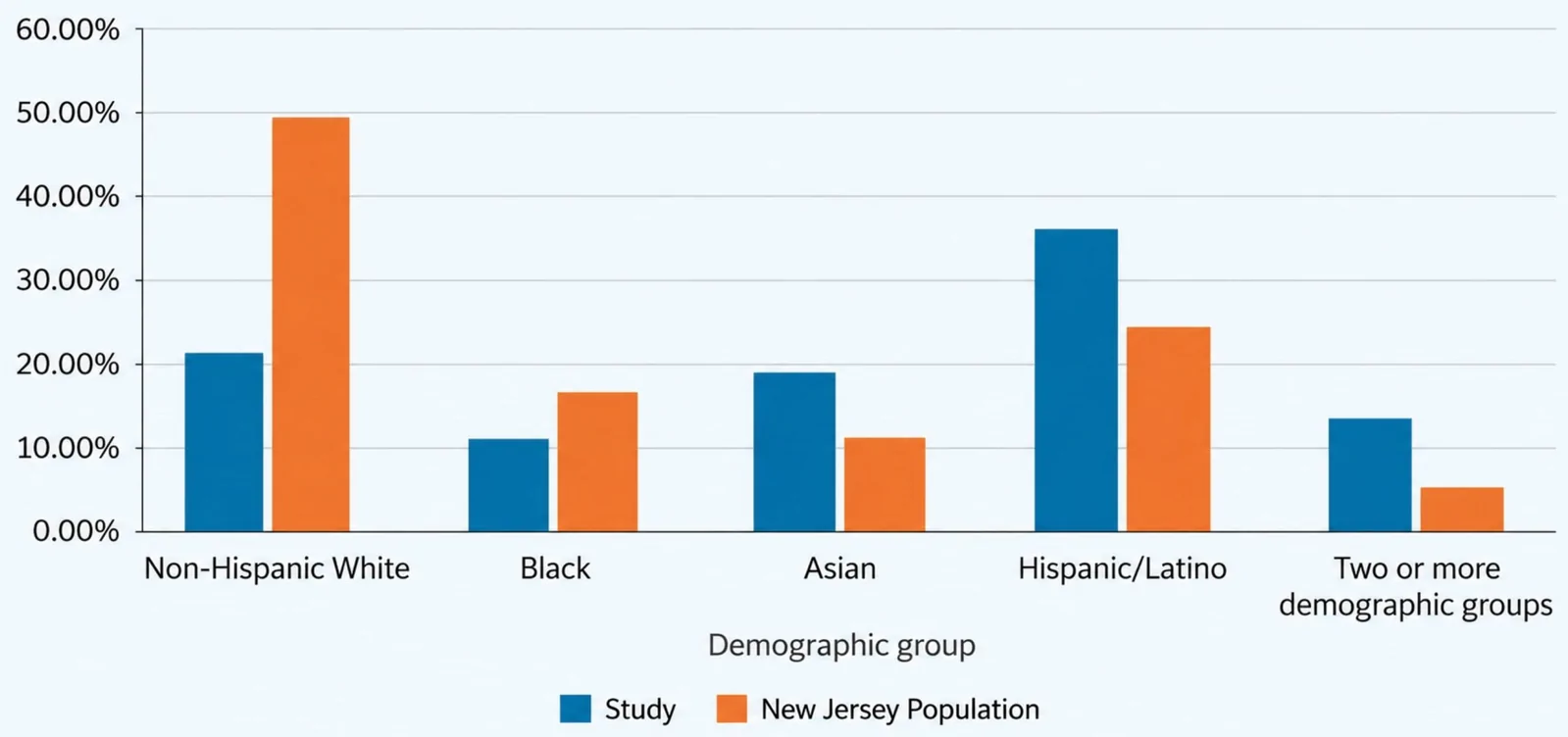
Demographic distribution in the study participants compared to the New Jersey census data.

We included 52 patients from four major hospital centers in New Jersey referred for RWGS over a three-year period. Figure 3 compares the demographic distribution of our cohort with census data for New Jersey.

This demographic composition suggests that Asian and Hispanic patients are overrepresented and that White and Black individuals are underrepresented compared to the reported demographic population prevalence in the State of New Jersey. This may reflect the specific demographic characteristics of the geographic catchment area in New Jersey for our study participants.

Patients were referred for RWGS using generally accepted, previously formulated criteria [7,8,9,10]. The following general referral criteria were used:

1. Life-threatening disorders or disorders that were likely to lead to severe disability if untreated;
2. No specific etiology was suspected based on personal or family history;
3. Current management was not satisfactory.

Targeted tests were performed prior to RWGS in 8 patients. Chromosomal microarray analyses were performed in 5 patients, panel DNA tests in 2 patients, and methylation testing for Prader–Willi syndrome or Beckwith–Wiedemann syndrome in one patient each. A total of 7 of these targeted tests were inconclusive. The RWGS helped confirm a genetic diagnosis in 4 of these patients. One targeted test was performed in parallel with the RWGS and was concordant for a positive genetic result (patient 4-5 in Table 3).

We included both parents in the study for 45 patients. In 6 patients, only the mother was available for testing. One patient had a proband-only test. All but one positive patient listed in Table 2 had both parents included in the test; only the patient’s mother was available (patient ID 2-7). This patient had a pathogenic variant in gene RYR1 that was not present in her mother.

### 4.2. Genomic Testing

#### Whole-Genome Sequencing and Variant Interpretation

All genomic testing was performed at the Rady Children’s Institute for Genomic Medicine (RCIGM) Clinical Genome Center, a College of American Pathologists (CAP)-accredited and Clinical Laboratory Improvement Amendments (CLIA)-certified laboratory. Genomic DNA was extracted from whole blood, and PCR-free libraries were prepared prior to whole-genome sequencing (WGS) on Illumina, San Diego, CA, USA, NovaSeq 6000 or NovaSeq X Plus instruments. Sequencing achieved a genome coverage of ≥30×. Sequence reads were aligned to the human reference genome (GRCh37/hg19 or GRCh38, according to the clinical protocol in effect at the time of testing), and single-nucleotide variants, insertions/deletions, copy number variants, structural variants, mitochondrial DNA variants, variants in non-coding genes, and the SMN1/SMN2 copy number were identified using the DRAGEN bioinformatics pipeline (Illumina; San Diego, CA, USA, v3.7–4.3). Additional analyses included repeat expansion and uniparental disomy detection using validated computational methods.

We annotated and prioritized variants using the Fabric Genomics Enterprise/GEM platform based on genotype, inheritance pattern, phenotype, and established gene-disease associations. We incorporated phenotypic information—including Human Phenotype Ontology (HPO) terms when available—into phenotype-driven analyses, and also performed phenotype-agnostic analyses. A multidisciplinary team of genome analysts, molecular geneticists, and genetic counselors interpreted variants in accordance with American College of Medical Genetics and Genomics/Association for Molecular Pathology (ACMG/AMP) standards and the Clinical Genome Resource [12,13].

Variants were classified as pathogenic, likely pathogenic, variants of uncertain significance (VUS), likely benign, or benign according to the ACMG/AMP criteria. Reported findings included pathogenic and likely pathogenic variants that explained or were likely related to the participant’s phenotype, as well as selected phenotype-relevant VUS when deemed clinically informative. Incidental findings, including secondary findings, were reported in accordance with ACMG recommendations [14] and RCIGM reporting policies when participants had not opted out of receiving such results.

Clinically reportable variants requiring confirmation were verified using orthogonal methods, including Sanger sequencing, multiplex ligation-dependent probe amplification, or other validated assays, as appropriate. Final clinical reports were issued to the ordering provider through the electronic medical record.

Test results were available within 5 days of receiving the test specimens.

## Figures and Tables

**Table 1:** Demographic distribution of the study cohort and the corresponding proportion of positive results.

Demographic	Percentage of Total Patients Included in the Study	Number	Positives	% Positive Findings
Non-Hispanic White	19.2	10	4	40.0
Black	11.5	6	1	16.7
Asian	19.2	10	4	40.0
Hispanic/Latino	36.6	19	6	31.5
2 or more demographic groups	13.5	7	4	57.1

## Data Availability

Data is unavailable due to privacy or ethical restrictions.
